# Comment on: Surveillance pouchoscopy for dysplasia: Cleveland Clinic Ileoanal Pouch Anastomosis Database

**DOI:** 10.1093/bjs/znab284

**Published:** 2021-08-04

**Authors:** M E W Derks, M Te Groen, F Hoentjen, L A A P Derikx

**Affiliations:** Department of Gastroenterology and Hepatology, Radboud University Medical Center, Nijmegen, The Netherlands

*Dear Editor*

We thank Lightner and colleagues for their study on surveillance pouchoscopy in ulcerative colitis patients. The authors reported that neoplasia following ileal pouch-anal anastomosis (IPAA) is rare. They recommended pouch surveillance with random biopsies of the anal transition zone (ATZ) for patients with a personal or family history of colorectal neoplasia (CRN)^1^.

It is unknown whether routine surveillance improves outcomes of pouch neoplasia, since pouch neoplasia is usually diagnosed after symptom development. As such, all patients in this study had a negative surveillance pouchoscopy in the 2 years prior to pouch carcinoma detection^1^. The authors refer to our nationwide cohort of 1200 IPAA patients including 16 with pouch carcinoma. Indeed, 13 of 16 patients in our cohort were diagnosed following symptoms leading to pouchoscopy. In contrast, dysplasia was detected in preceding surveillance pouchoscopies in only two of 11 patients with pouch carcinoma who underwent regular surveillance.

We question whether random biopsies lead to earlier detection of (pre)cancerous pouch lesions. The authors reported six pouch carcinomas and none were detected in random biopsies. Moreover, six of seven dysplastic lesions identified with random ATZ biopsies were not confirmed in follow-up biopsies and it could be questioned whether the initial lesions contained ‘true’ dysplasia^1^. In our cohort, four of 16 ATZ adenocarcinomas were diagnosed after random biopsies. However, these patients had prior CRN, which may warrant random ATZ biopsies in these high-risk patients.

Finally, we disagree with the recommendation of pouch surveillance in patients with a family history of CRN. The authors reported a positive family history in only one of 13 patients and previous studies did not identify this as a risk factor for pouch neoplasia.

In conclusion, we only advocate pouch surveillance with random ATZ biopsies in high-risk patients with prior CRN. Nevertheless, current data remain scarce and uniform, widely adopted pouch surveillance guidelines are needed to unify practice.

*Disclosure.* The authors declare no conflicts of interest.
